# A comparison of location of acute symptomatic vs. ‘silent’ small vessel lesions

**DOI:** 10.1111/ijs.12558

**Published:** 2015-06-29

**Authors:** Maria del C. Valdés Hernández, Lucy C. Maconick, Susana Muñoz Maniega, Xin Wang, Stewart Wiseman, Paul A. Armitage, Fergus N. Doubal, Stephen Makin, Cathie L. M. Sudlow, Martin S. Dennis, Ian J. Deary, Mark Bastin, Joanna M. Wardlaw

**Affiliations:** ^1^ Centre for Clinical Brain SciencesUniversity of EdinburghEdinburghUK; ^2^ Department of Cardiovascular SciencesUniversity of SheffieldSheffieldUK; ^3^ Institute of Genetics and Molecular MedicineUniversity of EdinburghEdinburghUK; ^4^ Centre for Cognitive Ageing and Cognitive EpidemiologyDepartment of PsychologyUniversity of EdinburghEdinburghUK

**Keywords:** acute lacunar infarct, lacunar stroke, lacunes, small vessel disease, stroke, white matter hyperintensities

## Abstract

**Background:**

Acute lacunar ischaemic stroke, white matter hyperintensities, and lacunes are all features of cerebral small vessel disease. It is unclear why some small vessel disease lesions present with acute stroke symptoms, whereas others typically do not.

**Aim:**

To test if lesion location could be one reason why some small vessel disease lesions present with acute stroke, whereas others accumulate covertly.

**Methods:**

We identified prospectively patients who presented with acute lacunar stroke symptoms with a recent small subcortical infarct confirmed on magnetic resonance diffusion imaging. We compared the distribution of the acute infarcts with that of white matter hyperintensity and lacunes using computational image mapping methods.

**Results:**

In 188 patients, mean age 67 ± standard deviation 12 years, the lesions that presented with acute lacunar ischaemic stroke were located in or near the main motor and sensory tracts in (descending order): posterior limb of the internal capsule (probability density 0·2/mm^3^), centrum semiovale (probability density = 0·15/mm^3^), medial lentiform nucleus/lateral thalamus (probability density = 0·09/mm^3^), and pons (probability density = 0·02/mm^3^). Most lacunes were in the lentiform nucleus (probability density = 0·01–0·04/mm^3^) or external capsule (probability density = 0·05/mm^3^). Most white matter hyperintensities were in centrum semiovale (except for the area affected by the acute symptomatic infarcts), external capsules, basal ganglia, and brainstem, with little overlap with the acute symptomatic infarcts (analysis of variance, *P* < 0·01).

**Conclusions:**

Lesions that present with acute lacunar ischaemic stroke symptoms may be more likely noticed by the patient through affecting the main motor and sensory tracts, whereas white matter hyperintensity and asymptomatic lacunes mainly affect other areas. Brain location could at least partly explain the symptomatic vs. covert development of small vessel disease.

## Introduction

One quarter of patients presenting with ischaemic stroke have ‘lacunar’ or ‘small vessel’ stroke 1. The responsible lesion is an acute lacunar (or recent small subcortical) infarct, which appears hyperintense on diffusion‐weighted magnetic resonance imaging (DWI‐MRI) 2. These recent small subcortical infarcts evolve over a few weeks to months to leave either a small cavity (lacune) or a small noncavitated hyperintense lesion on FLAIR or T2 that resembles a white matter hyperintensity (WMH) 3, 4, 5. Thus, long term, the appearance can be identical to the WMH and lacunes 6 that are commonly seen on MRI in older people scanned for other reasons but who have *never* experienced any stroke symptoms. New lacunes and WMH may also appear on follow‐up imaging in patients who have not noticed any new symptoms 3.

Recent small subcortical ischaemic stroke, WMH, and lacunes are part of the spectrum of small vessel disease (SVD) 7, 8. It is unclear why some SVD lesions cause the patient to present with stroke and so may lead to earlier diagnosis of SVD, whereas others, with very similar imaging appearances, accumulate covertly.

We tested the hypothesis that the location of SVD lesions in the brain determines, at least in part, why some cause acute lacunar stroke neurological symptoms, whereas others can develop in the brain covertly, even becoming quite numerous and leading to cognitive impairment, but without apparently causing discrete stroke symptoms that might lead the patient to seek medical attention earlier.

## Methods

### Subjects

*Stroke patients:* We used data from three prospective consecutive nonoverlapping studies of patients presenting with acute stroke to one regional hospital, who underwent MRI: Study 1 from 2002 to 2005 9, Study 2 from 2005 and 2007 10, and Study 3 from 2010 to 2013. From these studies, we selected all patients who had presented with an acute lacunar stroke clinical syndrome according to the Oxfordshire Community Stroke Project Classification which does not use risk factors to diagnose the stroke syndrome 11 (i.e. pure motor hemiparesis, pure sensory syndrome, sensorimotor syndrome, dysarthria‐clumsy hand syndrome, or ataxic hemiparesis), with a DWI‐MRI‐positive recent small subcortical infarct consistent with this index stroke less than 2‐cm maximum axial diameter and no past history of stroke: 87 patients from Study 1, 32 from Study 2, and 75 from Study 3 (total: 194). Patients with more than one DWI‐MRI positive lesion were excluded. All patients were assessed by experienced stroke physicians, and had baseline demographics, full medical history, examination, and investigations for stroke 9, 10. Written informed consent was obtained from all patients; all studies had Research Ethics Committee approval (Study 1, LREC2001/4/46; Study 2, LREC 2002/8/64; Study 3, LREC 09/S1101/54).

In order to map the corticospinal tracts from diffusion tensor imaging (DTI) and for age‐ and population‐relevant image registration purposes, we used data from 517 community‐dwelling older subjects aged 71–74 years without history or imaging evidence of stroke or incidental finding, the Lothian Birth Cohort 1936 (LBC1936) (www.lothianbirthcohort.ed.ac.uk/) 12. The presence of WMH alone was not excluding. Written informed consent was obtained from all participants. The study was approved by the Lothian (REC 07/MRE00/58) and Scottish Multicentre (MREC/01/0/56) Research Ethics Committees.

### MR brain image acquisition

All MRI data were acquired on the same 1.5T GE Signa Horizon HDxt MRI scanner (General Electric, Milwaukee, WI, USA) using self‐shielding gradients (maximum gradient 33 mT/m) and an eight‐channel phased‐array head coil. The scanner underwent daily quality assurance tests to maintain operational standards. The stroke studies had T1‐weighted (T1‐W) sagittal, T2‐W, Fluid Attenuated Inversion Recovery (FLAIR), gradient echo (GRE), and DWI axial imaging with virtually identical sequences 9, 10; the LBC1936 had T2‐W, fluid attenuation inversion recovery (FLAIR), T2*‐W axial, and T1 volume coronal MR images and DTI 12 (Table S1).

### Image processing

All image processing was performed blind to clinical details.

We masked the *recent small subcortical infarct* seen on DWI on the FLAIR image using Analyze 11.0^TM^ (http://www.analyzedirect.com/Analyze/). We used the FLAIR image for consistency of registration with the WMH maps and to avoid acute stroke lesion distortion from registration and blooming artifacts.

One trained observer segmented the *WMH* using a validated semiautomated technique, MCMxxxVI 13 (http://sourceforge.net/projects/bric1936/), a multispectral method that fuses two MRI sequences into the red/green/blue color space and performs minimum variance quantization to separate tissues and lesions. Any segmentation errors were corrected manually. The intraclass correlation coefficient was 0·964, *P* < 0·01, two‐tailed indicating excellent observer reliability 13. We use the term ‘WMH’ for convenience to include FLAIR hyperintensities in cerebral hemispheric white matter, deep grey matter, and brain stem 2.

We identified *lacunes* (round or ovoid cavities of CSF signal on FLAIR, T1‐W and T2‐W images with diameters ≥3 mm^2^) semi‐automatically by thresholding the FLAIR images using Analyze 11.0^TM^.

To compare SVD lesion locations, we aligned all brain images in standard space. We used the Mahalanobis distance 14 to derive the population‐representative ‘average’ brain from the LBC1936 cohort, that is, the subject whose intracranial, brain, ventricular, and WMH volumes were all closest to the cohort median (Method S1) 12, 15. We registered the representative LBC1936 subject's T1‐W volume and FLAIR images to standard space using the MNI‐152 isotropic T1‐W 1‐mm brain template and linear rigid body registration (http://fsl.fmrib.ox.ac.uk/fsl/fslwiki/FLIRT). We registered the individual stroke patients' or LBC1936 subjects' images and lesion masks to the representative brain in standard space using affine linear transformation. We mapped the stroke patients' recent small subcortical infarct, WMH, and lacune masks to the standard space brain. We summed the individual masks to generate group spatial probability density (PD) maps, where the PD in each voxel represents the proportion of the population with a recent small subcortical infarct, WMH, or lacune involving that voxel. Thus, if all patients had a WMH in the same voxel, the PD for WMH in that voxel would be 1; if half the population had a WMH in that voxel, the PD would be 0·5. Note that all patients contributed to the PD of recent small subcortical infarcts and WMH, but only 87 patients contributed to the PD of lacunes; thus, the PD of lacunes is calculated considering only the 87 patients who had them.

To quantify the anatomical location of the lesion probabilities, we generated a computational template of standard subcortical structures (Fig. S1) using FSL FIRST (http://www.fmrib.ox.ac.uk) for subcortical grey matter and a DTI white matter atlas 16 for standard major white matter tracts. We placed 2‐mm^3^ regions‐of‐interest on the standard structures and measured the PD in each. We checked the computationally determined PDs using manual region‐of‐interest placement (Supplementary Methods). We also extracted the corticospinal tracts from the LBC1936 Cohort using DTI data processed with probabilistic neighborhood tractography 17 (BedpostX/ProbTrackX algorithm) and TractoR software (http://www.tractor‐mri.org.uk/.17) excluding tracts with failed processing: the corticospinal tracts were reliably segmented in 507/517 (98%) subjects. Finally, to check for recent small subcortical infarct–WMH overlap, we subtracted the former from the latter masks.

### Statistical analysis

All parameters assessed were nonparametric; therefore, descriptive data are reported as medians and interquartile ranges (IQRs). We compared the lesion distributions using the Friedman's two‐way anova by ranks in IBM SPSS Statistics version 21 (IBM Corporation, Armonk, NY, USA; http://www‐01.ibm.com/software/analytics/spss/products/statistics/).

## Results

Image registration performed well for 192/194 stroke patients (99%), and FLAIR data were incomplete/absent for six patients and corrupted with severe artifacts on one; so, the WMH and recent small subcortical infarct maps use data from 188/194 patients and for lacunes from 187/194 patients. The characteristics of the stroke patients were similar in the three contributing stroke studies (Table 1). The median recent small subcortical infarct volume was 0·79 ml (IQR = 0·9). The median WMH volume was 17·4 ml (IQR = 29·6) in the stroke patients and 6·9 ml (IQR = 11·5) in the LBC1936 subjects (*P* < 0·001).

**Table 1:** Baseline characteristics of the patients with stroke and (for comparison) community‐dwelling older subjects without stroke

Original study	Stroke Study 1	Stroke Study 2	Stroke Study 3	Normal older subjects^c^
Total sample	*n* = 298^a^	*n* = 97^a^	*n* = 264^a^	*n* = 517
Patients with recent small subcortical infarct on DWI	87^b^	32	75	n/a
Age years (mean ± SD; range)	69 ± 12	64·5 ± 12·1; 36–91	65·9 ± 11·6; 39–96	72·7 ± 0·7; 71–74·2
Male (%)	44 (60)	21 (66)	48 (64)	273 (53)
Diabetes (%)	8 (9)	4 (14)	9 (12)	52 (10)
Hypertension (%)	41 (47)	20 (61)	60 (80)	244 (47)
History of cardiovascular disease (%)	17 (20)	3 (10)	12 (16)	139 (27)
Median WMH volume ml (IQR)	15·3 (25·1)	16·2 (28·1)	19·7 (34·0)	6·9 (11·5)
Median recent small subcortical infarct volume ml (IQR)	0·9 (1·1)	0·7 (0·7)	0·65 (0·8)	n/a
Number of lacunes	49	10	28	n/a
Median lacune volume ml (IQR) in those with lacunes	0·17 (0·27)	0·22 (0·16)	0·13 (0·22)	n/a

a Indicates the total number of patients in the original study including patients with nonlacunar stroke.

b Six of 87 patients lacked FLAIR images for WMH mapping and registration failed in two patients.

c These control subjects had never had a stroke.

n/a, not applicable.

**Figure 1:**
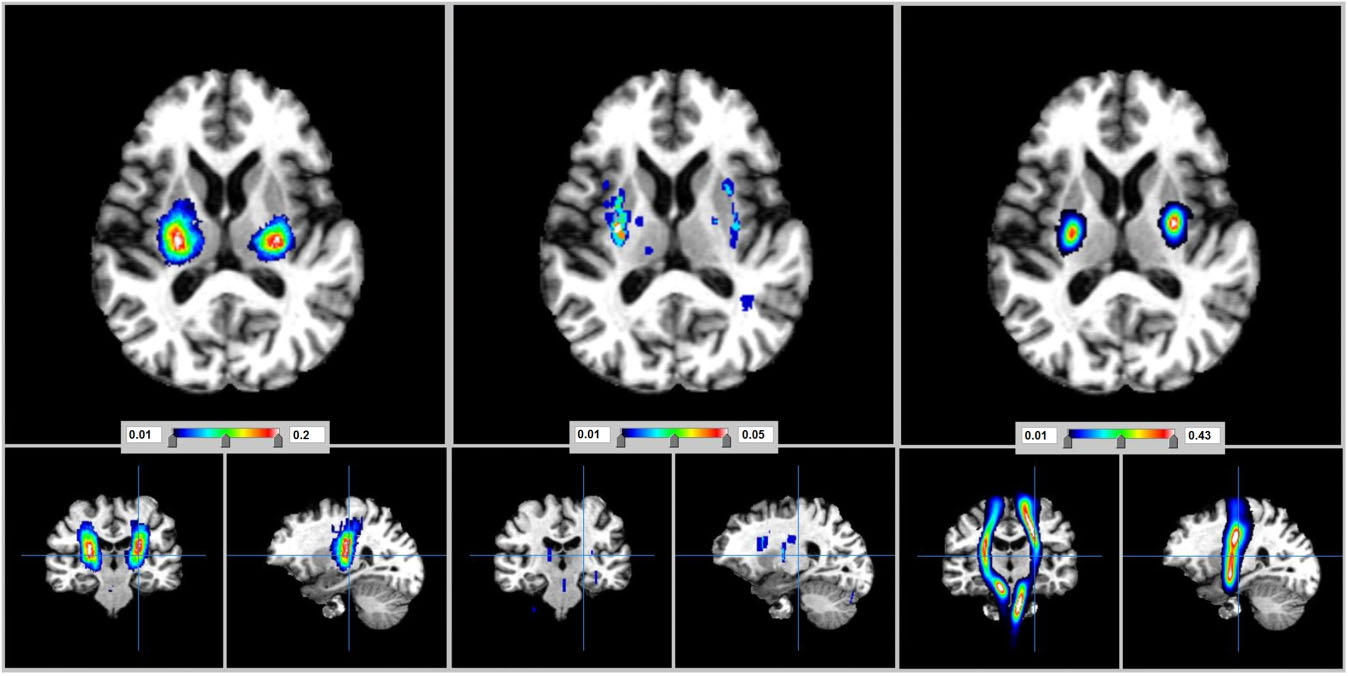
View in the axial, coronal, and sagittal planes to compare the distribution of recent small subcortical infarcts (left) and lacunes (middle) in stroke patients. The location of the corticospinal tracts as derived from tractography (right) is shown for comparison. Also, see Fig. S2.

The commonest location of the recent small subcortical infarcts was in the superior aspect of the mid‐posterior limb of the internal capsule (PD 0·2/mm^3^) (Fig. 1, Table 2). The probability progressively decreased from there medially to the lateral thalamus (PD 0·17/mm^3^), superiorly to the corona radiata adjacent to the body of the lateral ventricle (PD 0·15/mm^3^), laterally to the medial–posterior aspect of lentiform nucleus (PD 0·08/mm^3^), and inferiorly to the midbrain/superior brainstem (PD 0·05–0·07/mm^3^). Comparison with the DTI‐derived corticospinal tract map (Fig. 1) showed that the recent small subcortical infarcts occurred almost exclusively in the motor or sensory corticospinal tracts.

**Table 2:** Probability of finding recent small subcortical infarcts, WMH, or lacunes in each brain region in patients with stroke, and of finding WMH in the community‐dwelling older subjects without stroke (for comparison with the WMH in stroke patients)

Brain region^†^	Probability density of voxels in each region being affected by the SVD lesions							
	Patients with stroke						Normal older subjects	
	Recent small subcortical infarct^‡^		Lacunes		WMH		WMH	
	Right	Left	Right	Left	Right	Left	Right	Left
Caudate	0·05	0·12	0·01	0·01	0·56	0·54	0·49	0·49
Putamen	0·16	0·15	0·03	0·05	0·18	0·22	0·09	0·11
Globus pallidus	0·09	0·11	0·02	0·04	0·16	0·12	0·10	0·08
Thalamus	0·14	0·15	0·005	0·005	0·20	0·26	0·18	0·26
Hippocampus	0·005	0·005	0·01	0·01	0·09	0·14	0·14	0·17
Superior aspect of posterior limb of internal capsule *1*	0·2	0·2	0·05	0·05	0·1	0·15	0·01	0·01
Mid posterior limb internal capsule and adjacent lateral thalamus and medial lentiform nucleus *2*	0·17	0·17	0·04	0·04	0·14	0·14	0·07	0·07
Corona radiata lateral to body of lateral ventricle *3*	0·15	0·15	0·005	0·005	0·39	0·43	0·11	0·11
Corona radiata superior to lateral ventricle *4*	0·14	0·14	0·005	0·005	0·57	0·53	0·57	0·57
Medial lentiform nucleus *5*	0·06	0·08	0·005	0·005	0·02	0·02	0·01	0·01
Centrum semiovale	0·02	0·02	0·005	0·005	0·62	0·62	0·6	0·6
Retro‐lentiform part of the internal capsules	0·02	0·02	0·04	0·04	0·26	0·26	0·12	0·12
Anterior limb internal capsule *6*	0	0·05	0·005	0	0·05	0·05	0·02	0·07
Adjacent to anterior horns of the lateral ventricles	0	0	0·005	0·005	0·77	0·77	0·70	0·70
Optic radiations	0	0	0	0·005	0·55	0·49	0·47	0·38
External capsules	0·1	0·1	0·05	0·05	0·25	0·25	0·12	0·12
Midbrain/superior brainstem* *7*	0·07		0·005		0·02		0·01	
Brainstem*	0·02		0·005		0·14		0·03	

The probability density refers to the proportion of voxels in the particular region‐of‐interest that had a lesion in the stroke patients or (for WMH) in the community‐dwelling older subjects without stroke. For example, a probability of 0·005 (or 1/188) indicates that only one subject out of the 188 stroke patients had a voxel affected by a lesion in this region. These are also adjusted by the number of individuals, within the population, that contributed data to the probability map. For example, whereas the whole stroke population had recent small subcortical infarct lesion(s), only 87 had lacunes. The probability density of lacunes is calculated considering only the 87 patients who had them. *Structure not separated into right and left due to small size.

^†^Numbers in italics refer to region numbers in Fig. S1. ^‡^As recent small subcortical infarcts were largely confined to the primary motor/sensory pathways, their median probability value across all subcortical regions was 0, and their mean probability value and standard deviation were of the orders of 10^−6^ and 10^−4^, respectively.

**Figure 2:**
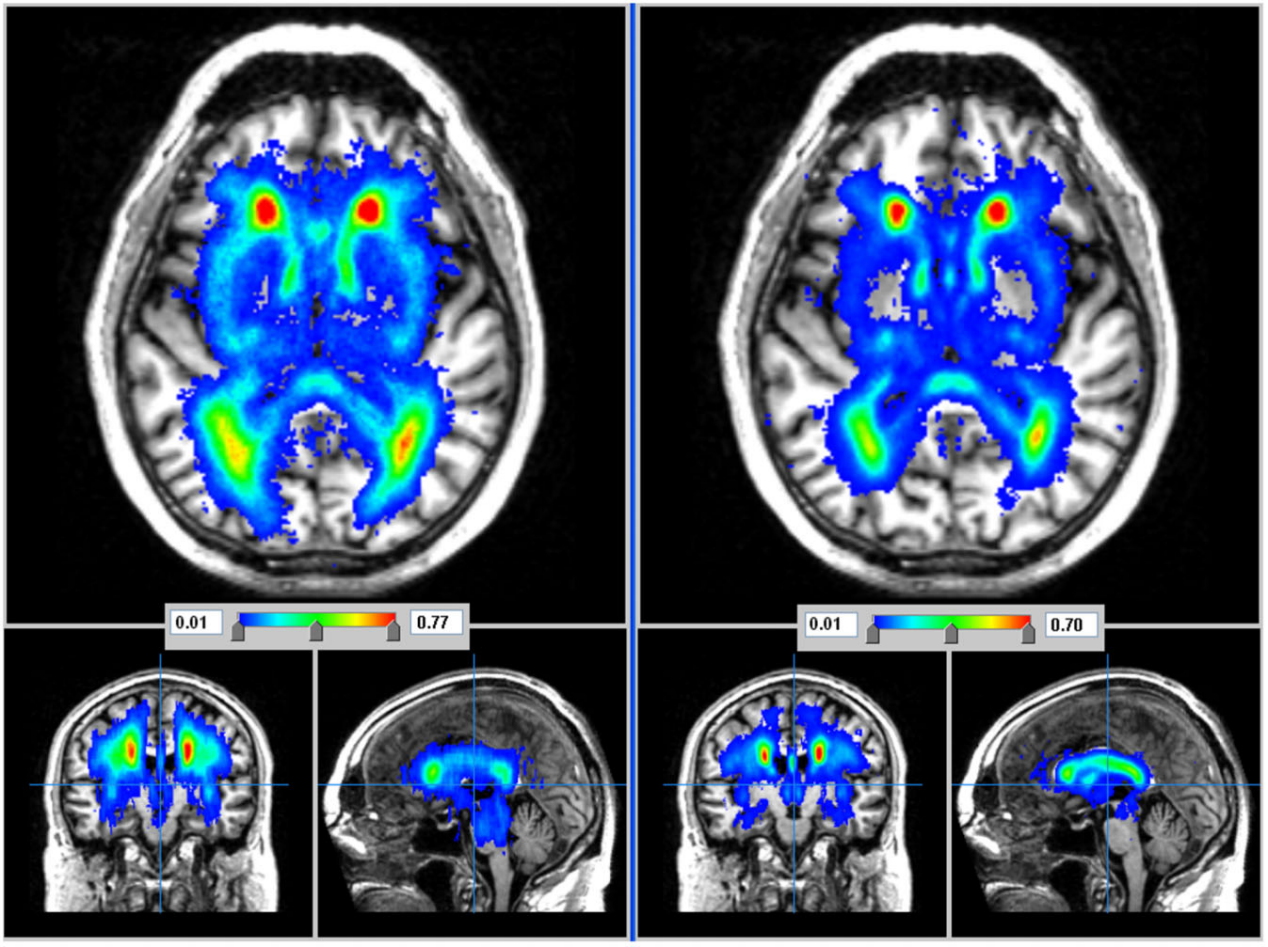
WMH distribution of 188 stroke patients (left) and (for comparison) in 517 similarly aged subjects without stroke (right) in axial, coronal, and sagittal planes. Burden of WMH is less in stroke‐free subjects although the distribution is similar. See also Fig. S3.

**Figure 3:**
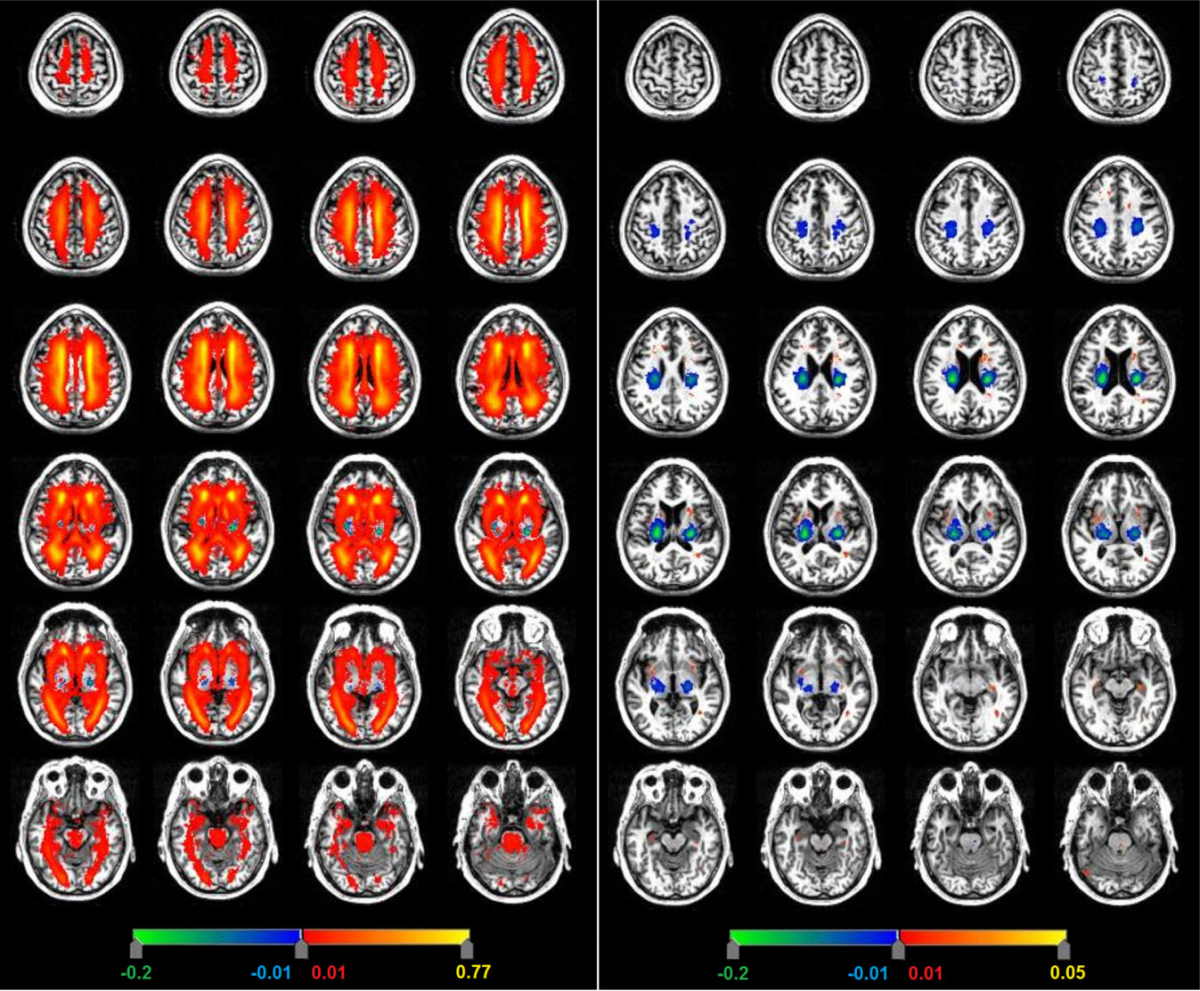
Subtraction image to determine the difference in distribution of the recent small subcortical infarcts with respect to WMH (left) and lacunes (right) in stroke patients. Red–yellow indicates WMH (left hand image) or lacunes (right hand image); green–blue regions indicate the recent small subcortical infarcts that remain after subtraction from WMH and lacune maps (both sides). Images show a little overlap in location between the clinically symptomatic vs. covert lesions.

In patients with stroke, the WMH were distributed symmetrically between the hemispheres in white matter, in external capsules, basal ganglia, and brainstem (Fig. 2) but were infrequent where the recent small subcortical infarcts were most frequent (anova, *P* < 0·01; Table 2). Subtraction of the recent small subcortical infarcts from the WMH map confirmed that there was little overlap in location (Fig. 3). The WMH *anatomical distribution* was similar in the stroke patients and the LBC1936 subjects (Fig. 2, Table 2), although WMHs were *more extensive* in the stroke patients (ANOVA, *P* < 0·008; also Fig. S3).

Lacunes were identified in 87/187 stroke patients (Figs 1 and S2), median volume 0·14 ml (IQR 0·19 ml, range 0·03–0·8 ml; Table 1). Lacunes were most frequent in the posterior external capsules (PD 0·05/mm^3^) and lateral parts of posterior lentiform nuclei (PD 0·04/mm^3^), that is, lateral to the most frequent location of the recent small subcortical infarcts. Lacunes were also present in the caudate (PD 0·01/mm^3^), hippocampus (PD 0·01/mm^3^), and in parts of thalamus (PD 0·005/mm^3^) where no acute symptomatic lesions occurred (difference in distribution of acute SSI and lacunes ANOVA, *P* < 0·03; Fig. 3).

## Discussion

The distribution of the three SVD lesion types indicates that the lesions that present with acute stroke symptoms are located mostly in the corticospinal tracts, particularly in the posterior limb of the internal capsule. Here, perhaps even a small lesion can cause symptoms because interruption of primary motor or sensory function where the tracts are tightly packed would readily be noticed by the patient. In contrast, the WMH and lacunes, which had never caused discrete symptoms, were more common in other subcortical regions where interruption of some fibers might be less likely to be noticed by the patient. This could explain why recent small subcortical infarcts come rapidly to medical attention through causing a ‘stroke’, whereas many WMH and lacunes can accumulate over time without individually causing enough symptoms for the patient to seek medical attention until sufficient brain damage has occurred to trigger presentation with cognitive or mobility problems. Alternatively, if seeking medical attention, their symptoms may not be recognized as of importance 18, 19.

We included almost 200 patients with acute lacunar stroke symptoms and a DWI‐visible acute lesion to ensure correct identification of the index stroke, all recruited prospectively. We used voxel‐based region‐of‐interest sampling on a purpose‐designed standard anatomical template that represents subcortical white and grey matter. We used a study‐specific population‐relevant brain template from subjects scanned on the same scanner to optimize image registration and minimize lesion distortion. However, we did not examine lesion distribution of individual lacunar stroke syndromes as our main purpose was to compare SVD lesions that presented with stroke to those that did not – such an analysis of subtypes of lacunar syndrome would require a larger sample. We did not find any DWI‐positive lesions that were asymptomatic as have been seen in some patients with recent small subcortical infarcts 20 or with recent cerebral hemorrhage 21. However, in our experience and the literature, these asymptomatic DWI positive small subcortical infarcts are infrequent, perhaps present in less than 5% of patients, and therefore, it will take some time to accumulate enough subjects, even in multicentre studies, to be able to plot their distribution reliably. Serial longitudinal studies with detailed symptom ascertainment and repeat scanning will be required to examine symptomatology as new lesions accumulate and in individual lacunar subsyndromes. Nor did we have long‐term follow‐up images on our patients to see if any lacunes that resulted from late‐stage cavitation of any of the recent small subcortical infarcts were of different size to the asymptomatic lacunes, as another possible explanation for a difference in symptoms. However, C Miller Fisher and others did not find that size of lacunes measured at post mortem was related to symptoms 22, 23. Further long‐term follow‐up is required to address this question.

Our findings support: the findings of C Miller Fisher's meticulous dissections suggesting that lesions in the internal capsule were more likely to have caused symptoms in life than those in other brain regions 22, and by other limited post mortem data 23; patients with CADASIL where asymptomatic lacunes affected the thalamus and lentiform nucleus 24; and a previous study using T2‐weighted MR showing that lesions associated with stroke symptoms were more often in the internal capsule posterior limb and adjacent basal ganglia than were similar‐appearing lesions in patients without stroke 25. The different distribution of the lacunes and WMH in these sporadic SVD patients is also not dissimilar to differences in lacunes and WMH distributions noted previously in patients with CADASIL 26. WMH volume of the stroke patients was two to three times that of the stroke‐free LBC1936 subjects, despite the similar median age, perhaps reflecting the higher prevalence of hypertension in stroke patients. A higher WMH burden in stroke patients than in subjects without stroke has also been noted previously 27, 28.

Other factors may explain why some SVD lesions cause stroke symptoms while others develop covertly. Speed of onset (e.g. WMH) may develop more gradually than recent small subcortical infarcts. Severity of tissue damage (e.g. recent small subcortical infarcts) may be more destructive to the tissue than many WMH, as suggested by the former's higher rate of cavity formation 3. Multidirectionality of fibres (e.g. centrum semiovale white matter fibers) is more multidirectional than in the internal capsule, possibly ‘diluting’ the impact of a small lesion across several functions. Size (e.g. WMH) may be smaller than recent small subcortical infarcts, although the range of recent small subcortical infarcts includes lesions of 5 mm or less 29. Recent reports indicate that WMHs are associated with awareness of cognitive decline 19 and subtle physical symptoms 18, 30, suggesting that they are not as ‘silent’ as previously thought. Differences in size of lacune and symptoms require evaluation in further studies, as just discussed. Meanwhile, our anatomical analysis provides further evidence that presentation with an acute lacunar stroke heralds a diffuse brain disease 7, 8 and provides methods to map subcortical structures for future detailed longitudinal studies.

## Supporting information


**Table S1.** MR imaging sequence details for the cohorts analysed in this work.
**Fig. S1.** Representative brain showing the subcortical grey matter structures, indicated in orange and yellow in the left and right hemispheres respectively, and the standard sampling points (numbered red dots) used to measure the PD of recent small subcortical infarcts.
**Fig. S2.** Slice‐by‐slice distribution of recent small subcortical infarcts (left) and lacunes (right) in patients with stroke.
**Fig. S3.** WMH in patients with stroke (left) and in 517 community‐dwelling older subjects without stroke (right).
**Method S1.** A comparison of brain location of acute symptomatic infarcts vs. ‘silent’ small vessel lesions.
