# Supplementary material for: A comparison of location of acute symptomatic vs. ‘silent’ small vessel lesions
Source: Int J Stroke. 2015 Jun 29;10(7):1044–50. doi: 10.1111/ijs.12558 (PMC4737263; doi:10.1111/ijs.12558)
Supplement: Supplementary file 1 — Table S1. MR imaging sequence details for the cohorts analysed in this work. Fig. S1. Representative brain showing the subcortical grey matter structures, indicated in orange and yellow in the left and right hemispheres respectively, and the standard sampling points (numbered red dots) used to measure the PD of recent small subcortical infarcts. Fig. S2. Slice‐by‐slice distribution of recent small subcortical infarcts (left) and lacunes (right) in patients with stroke. Fig. S3. WMH in patients with stroke (left) and in 517 community‐dwelling older subjects without stroke (right). Method S1. A comparison of brain location of acute symptomatic infarcts vs. ‘silent’ small vessel lesions. [file IJS-10-1044-s001.doc]

**A comparison of brain location of acute symptomatic infarcts versus ‘silent’ small vessel lesions**

**Supplementary methods and tables**

*Derivation of a representative brain for image registration and lesion mapping in standard space:* The importance of using a relevant population-based brain template has been stressed.^1^ In previous work, we found that the average brain templates of young individuals (e.g. MNI-152), whose head sizes are larger than our older subjects, causes significant image distortion due to registration effects. Instead, the use of a single brain that is representative of the study population has been tested and accepted as valid for distributional mapping and spatial normalisation.^2,3^

We selected the representative brain from the LBC1936 group mathematically using the Mahalanobis distance, using head size, superficial cortical and ventricular atrophy, and spatial intensity distribution criteria as follows.^4^ We used volumetric measurements of the intracranial volume (ICV) and brain tissue, ventricles (each separately) and subarachnoid space, normalised to ICV, and of white matter hyperintensities (WMH) normalised to brain volume. We extracted the ICV automatically using the GRE sequence and the Object Extraction Tool in Analyze^TM^ 10.0 (AnalyzeDirect Inc, Mayo Clinic), with manual editing when required.^5^ Ventricles were extracted and quantified on the T1-weighted (T1-W) volumes using the thresholding region-growing algorithm in Analyze^TM^ 10.0. The threshold was set as the mean intensity between the lateral ventricle and the superior temporal cortex. The other measurements used the MCMxxxVI tool as explained previousl. We calculated the mean and variance of all normalised volumetric measurements in standard units for each brain. We added the standard units for each subject to calculate the Mahalanobis distance and listed the subjects in ascending order. The subject with smallest Mahalanobis distance (i.e. standard unit measurements closed to the mean of the population) was selected as the representative brain.

*Lesion distribution quantification:* We examined several methods to quantify the anatomical lesion probability density (PD) because all available anatomical segmentation tools had limitations. It was also important to avoid lesion distortion that can occur if warping individual brains into standard space,^6^ thus it was better to use native space. For example, FSL FIRST, a model-based subcortical grey matter segmentation tool from the FMRIB Software Library,^7^ segments only deep grey matter structures, but many recent small subcortical infarcts and most WMH are in white matter. A full brain parcellation based on the Talairach-Turnoix atlas, which includes 1011 structures implemented by FreeSurfer *(*<https://surfer.nmr.mgh.harvard.edu/>*),* focuses on cortical gyri, has not been reported as reliable for use in stroke patients^8^ and is unstable if measurements are obtained using different sequence parameters.^9^ Therefore, we used two complementary approaches. First, we developed a structured template (Supplementary Figure 1) based on a reference atlas textbook^10^ to establish standard subcortical regions of interest. Then we automated the template for assessing the regions of interest using subcortical grey matter maps determined using FSL FIRST and five main white matter pathways determined using the Johns Hopkins University diffusion tensor MRI (DT-MRI) white matter atlas (anterior horns of the lateral ventricles, optic radiations, centrum semiovale bordering the corona radiata, external capsules and retrolentiform part of the internal capsules).^11^ We placed 2 x 2 x 2mm ROI on the subcortical grey and white matter anatomical regions as indicated in Supplementary Figure 1 and then determined the maximum PD at each computationally determined ROI. Secondly, we checked the accuracy of the computational template by placing 1 mm^3^ isovoxels manually in standard brain regions on the PD lesion maps, guided visually by the region of interest template and the reference atlas.^10^

Supplementary Table 1. MR imaging sequence details for the cohorts analysed in this work.

| Study | Stroke Study 1  (protocol 1) | Stroke Study 2  (protocol 2) | Stroke Study 3 | LBC1936 Study |  |
| --- | --- | --- | --- | --- | --- |
| TR/TE/TI (ms) T1W | 440/9 | | 9.7/3.984/500 | |  |
| TR/TE (ms) DT-MRI | 6300/106.13 | 6300/107.64 | 10000/95.5 | 16500/95.5 |  |
| TR/TE (ms) T2*W | 2000/9.912 | 620/15 | 625/15 | 940/15 |  |
| TR/TE/ TI (ms) FLAIR | 10002/147/ 2500 | 9002/147/2200 | 9000/140/2200 | |  |
| Pixel bandwidth (KHz) | 125 (T1W)  81.38 (T2*W)  122.07(FLAIR) | 125 (T1W)  97.656(T2*W)  122.07(FLAIR) | 15.63 (T1-W,FLAIR)  12.5 (GRE) | |  |
| Matrix | 256x256 | 256x192 | 256x216 (T1W)  128x128 (DTI)  384x224 (T2*W, FLAIR) | 192x192 (T1-W)  128x128 (DT-MRI)  256x192(GRE)  256*256(FLAIR) | |
| No. slices | 19 | 20 | 256 (T1W)  28 (FLAIR,T2*W, T2W) | 160 (T1-W)  72 (DT-MRI)  80 (GRE)  40 (FLAIR) |  |
| Slice thickness (mm) | 5 | 5 | 1.02 (T1W)  5 (DTI,T2*W, FLAIR) | 1.3 (T1-W)  2 (DT-MRI,GRE)  4 (FLAIR) |  |
| Inter-slice gap (mm) | 1.5 | 1.5 | 1 | 0 |  |
| Voxel size (mm^3^) | 0.94x0.94x6.5 | 0.94x0.94x6.5 | 1.02x0.9x1.02 (T1W)  0.47x0.47x6 (T2-W,FLAIR, GRE) | 1.3x1.3x1(T1-W)  2x2x2 (DT-MRI)  1x1x2 (GRE)  1x1x4 (FLAIR) |  |

Supplementary Figure 1. Representative brain showing the subcortical grey matter structures, indicated in orange and yellow in the left and right hemispheres respectively, and the standard sampling points (numbered red dots) used to measure the PD of recent small subcortical infarcts. Numbers correspond to the regions with the highest to lowest PD of occurrence of recent small subcortical infarcts in descending order: superior aspect of the posterior limb of the internal capsule (1), lateral thalamus adjacent to posterior limb internal capsule (2), the corona radiata adjacent to the body of the inferolateral ventricle (3), corona radiata (4), medial lentiform nucleus (5), anterior limb of the internal capsule (6), midbrain/superior brainstem (7).


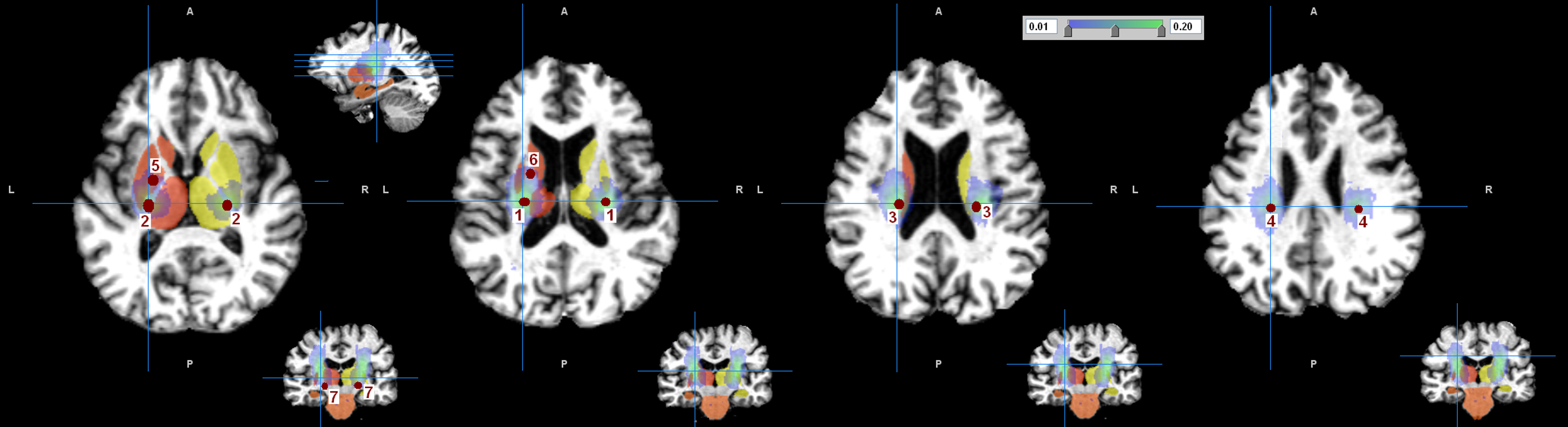


Supplementary Figure 2. Slice-by-slice distribution of recent small subcortical infarcts (left) and lacunes (right) in patients with stroke


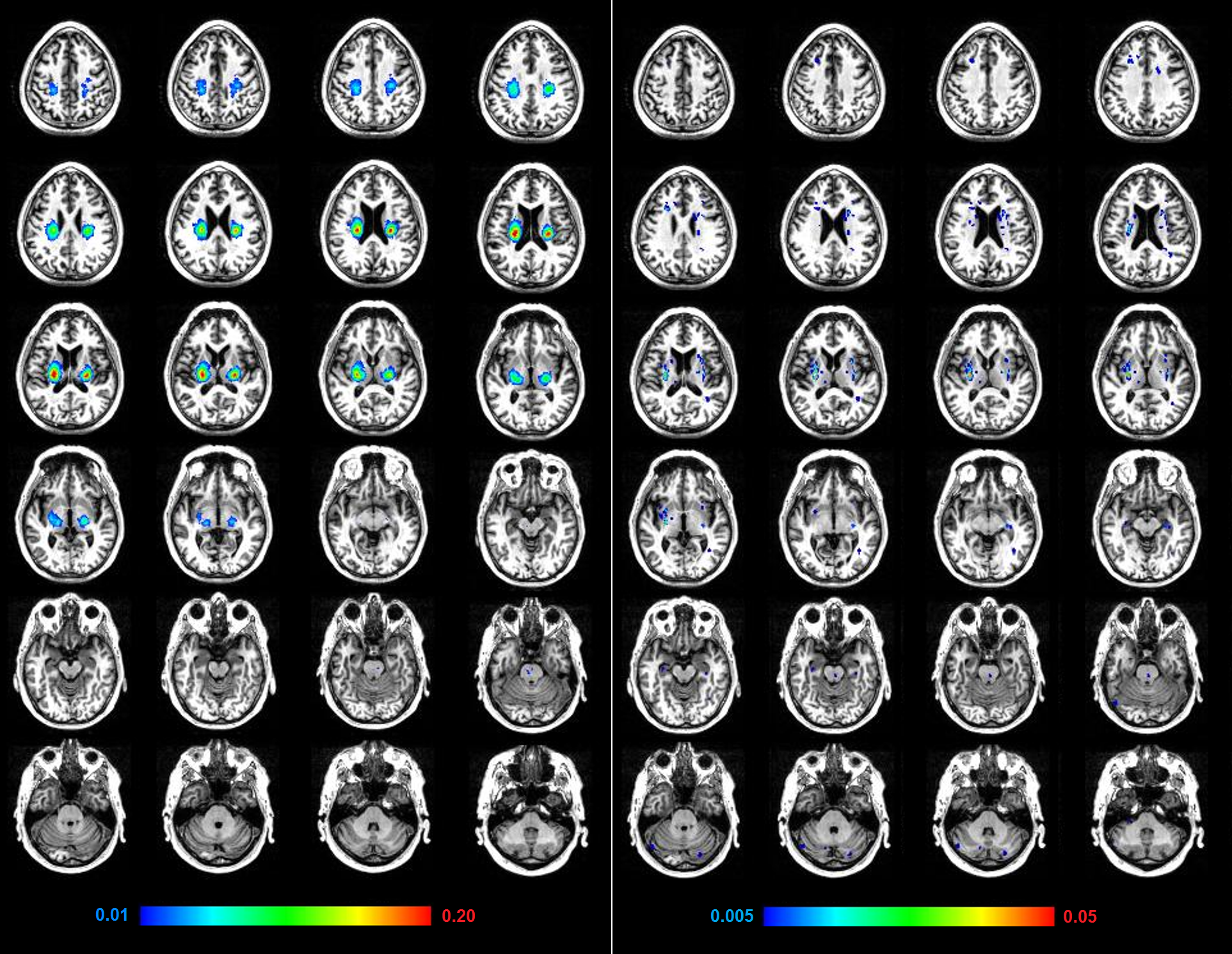


Supplementary Figure 3. WMH in patients with stroke (left) and in 517 community-dwelling older subjects without stroke (right). Note the similar distribution but lesser overall amount of WMH in the community-dwelling older subjects without stroke.


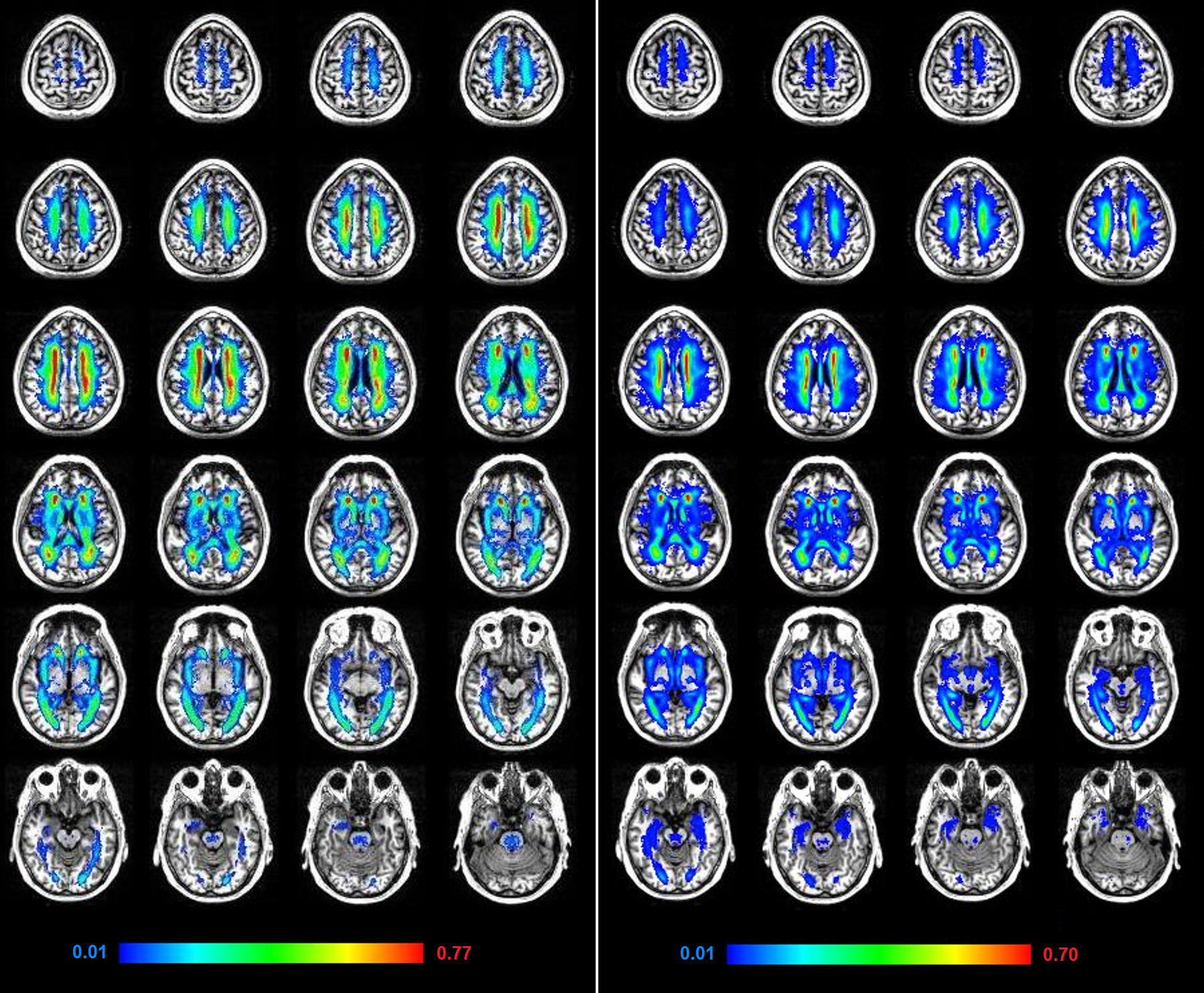


Reference List

1. Thompson PM, Woods RP, Mega MS, Toga AW. Mathematical/computational challenges in creating deformable and probabilistic atlases of the human brain. Hum Brain Mapp 2000;9:81-92.

2. Baillard C, Hellier P, Barillot C. Segmentation of brain 3D MR images using level sets and dense registration. Med Image Anal 2001;5:185-194.

3. Rohlfing T, Brandt R, Menzel R, Maurer CR, Jr. Evaluation of atlas selection strategies for atlas-based image segmentation with application to confocal microscopy images of bee brains. Neuroimage 2004;21:1428-1442.

4. De Maesschalck R, Jouan-Rimbaud D, Massart DL. The Mahalanobis distance. Chemometr Intell Lab Syst 2000;50:1-18.

5. Valdes Hernandez MC, Royle NA, Jackson MR, et al. Color fusion of magnetic resonance images improves intracranial volume measurement in studies of aging. Open Journal of Radiology 2012;2:1-9.

6. Aribisala BS, He J, Blamire AM. Comparative study of standard space and real space analysis of quantitative MR brain data. J Magn Reson Imaging 2011;33:1503-1509.

7. Patenaude B, Smith SM, Kennedy DN, Jenkinson M. A Bayesian model of shape and appearance for subcortical brain segmentation. Neuroimage 2011;56:907-922.

8. Sanchez-Benavides G, Gomez-Anson B, Sainz A, Vives Y, Delfino M, Pena-Casanova J. Manual validation of FreeSurfer's automated hippocampal segmentation in normal aging, mild cognitive impairment, and Alzheimer Disease subjects. Psychiatry Res 2010;181:219-225.

9. Jovicich J, Czanner S, Han X, et al. MRI-derived measurements of human subcortical, ventricular and intracranial brain volumes: reliability effects of scan sessions, acquisition sequences, data analyses, scanner upgrade, scanner vendors and field strengths. Neuroimage 2009;46:177-192.

10. Duvernoy HM. The Human Brain: Surface, Blood Supply, and Three-dimensional Anatomy, 2nd ed. New York, NY: Springer-Verlag, 1999.

11. Oishi K, Zilles K, Amunts K, et al. Human brain white matter atlas: identification and assignment of common anatomical structures in superficial white matter. Neuroimage 2008;43:447-457.
